# The Interplay between Histamine H_4_ Receptor and the Kidney Function: The Lesson from H_4_ Receptor Knockout Mice

**DOI:** 10.3390/biom11101517

**Published:** 2021-10-15

**Authors:** Roberta Verta, Maura Gurrieri, Sara Borga, Elisa Benetti, Paolo Pollicino, Roberta Cavalli, Robin L. Thurmond, Paul L. Chazot, Alessandro Pini, Arianna Carolina Rosa, Cristina Grange

**Affiliations:** 1 Department of Biotechnology and Health Sciences, University of Turin, C.So Dogliotti 14, 10126 Turin, Italy; roberta.verta@unito.it; 2 Department of Scienza e Tecnologia del Farmaco, University of Turin, Via P. Giuria 9, 10125 Turin, Italy; maura.gurrieri@gmail.com (M.G.); sara.borga@outlook.it (S.B.); elisa.benetti@unito.it (E.B.); roberta.cavalli@unito.it (R.C.); 3 Direzione Ricerca e Terza Missione, University of Turin, Via Bogino 9 Torino, 10123 Turin, Italy; paolo.pollicino@unito.it; 4 Janssen Research & Development, LLC, 3210 Merryfield Row, San Diego, CA 92121, USA; RTHURMON@its.jnj.com; 5 Department of Biosciences and Wolfson Research Institute, Durham University, South Road, Durham DH1 3LE, UK; paul.chazot@durham.ac.uk; 6 Department of Clinical and Experimental Medicine, University of Florence, Viale Pieraccini 6, 50139 Florence, Italy; alessandro.pini@unifi.it; 7 Department of Medical Sciences, University of Turin, C.So Dogliotti 14, 10126 Turin, Italy; cristina.grange@unito.it

**Keywords:** histamine H_4_ receptors, renal function, diabetic nephropathy

## Abstract

Previous studies implicated the histamine H_4_ receptor in renal pathophysiology. The aim here is to elucidate the role of this receptor on renal function using H_4_ receptor knockout mice (H_4_R^−/−^). Healthy and diabetic H_4_R^−/−^ mice compared to their C57BL/6J wild-type counterpart for renal function and the expression of crucial tubular proteins. H_4_R^−/−^ and wild-type mice, matched for ages, showed comparable weight gain curves reaching similar median weight at the end of the study. However, H_4_R^−/−^ mice displayed a higher basal glycemia. H_4_R^−/−^ mice showed a lower urine 24 h outflow, and albumin-to-creatinine ratio (ACR) compared to wild-type mice. Consistently, H_4_R^−/−^ mice presented a higher expression of megalin and a lower basal expression of the sodium-hydrogen exchanger (NHE)3 and aquaporin (AQP)2. According to these basal differences, diabetic H_4_R^−/−^ mice developed more severe hyperglycemia and a higher 24 h urine volume, but a lower increase in ACR and decrease in urine pH were observed. These events were paralleled by a reduced NHE3 over-expression and megalin loss in diabetic H_4_R^−/−^ mice. The AQP1 and AQP7 patterns were also different between H_4_R^−/−^ and wild-type diabetic mice. The collected results highlight the role of the histamine H_4_ receptor in the control of renal reabsorption processes, particularly albumin uptake.

## 1. Introduction

Histamine H_4_ receptor is the most recently discovered histamine receptor. Since its discovery in 2000 [1,2,3], histamine H_4_ receptor’s primary function as immunomodulatory was recognized [4,5] consistently with its expression in mast cells, eosinophils, neutrophils, and basophils [6]. However, additional evidence of a wider distribution of these receptors has been reported. In particular, since 2013, it has been demonstrated that the H_4_ receptor is expressed in the kidney [7]. The previous in vitro and ex vivo studies demonstrated that the H_4_ receptor is localized on the proximal tubules, thus suggesting it could participate in renal patho-physiology. Consistently, our group demonstrated that the H_4_ receptor antagonist JNJ-39758979 prevents renal damage in a mouse model of diabetes-induced nephropathy [8]. In particular, the data obtained suggested that histamine through the H_4_ receptor could exert both an indirect effect on renal tissue architecture by recruiting pro-inflammatory cells and, more importantly, direct modulation of tubular reabsorption. However, the histamine receptor subtype ligands’ specificity, efficacy and potency are a source of concerns [5]. Therefore, genetic knockout models are fundamental to understand the role of this specific histamine receptor subtype in kidney function and differentiate between off-target and on-target histamine H_4_ receptor effects. 

Histamine H_4_ receptor knockout (H_4_R^−/−^) mice were generated soon after the histamine H_4_ receptor discovery [9] and initially used to demonstrate the role of the histamine H_4_ receptor in mediating mast cells’ chemotaxis. Since then, H_4_R^−/−^ mice have been widely used to highlight the complexity of histamine H_4_ receptor function in allergy [10,11,12,13,14], inflammation [5], pain [15], or cancer [16]. However, to the best of our knowledge, none of these studies investigated the renal function in H_4_R^−/−^ mice. Nevertheless, although histamine was suggested to be involved in the etiopathology of diabetes complications [17], nephropathy in particular [18], no study to date involved diabetic H_4_R^−/−^ mice and evaluated their susceptibility to developing diabetic nephropathy. Therefore, the present study aims to clarify the functional role of renal histamine H_4_ receptor and its involvement in renal pathophysiology through the phenotypic characterization of healthy or diabetic H_4_R^−/−^ mice.

## 2. Materials and Methods

### 2.1. Materials

All chemicals, not otherwise indicated and rabbit polyclonal anti-β-actin antibody (A2066), were from Sigma Aldrich (St. Louis, MO, USA). The Glucocard MX Blood Glucose Meter was from A. Menarini Diagnostic (Florence, Italy). The Albumin enzymatic immunoassay kits ELISA Quantification Set (E90-134) was from Bethyl Laboratories Inc. (Montgomery, TX, USA). The Urine Strips were from GIMA S.p.a. (Gessate, MI, Italy). The Mouse interleukin (IL)-6 Quantikine ELISA Kit (M6000B) was from R&D Systems (Minneapolis, MN, USA). The goat polyclonal anti-megalin (P-20; sc-515750), anti-histamine H_2_ receptor (A20, sc-19773) and anti-Na-K-Cl cotransporter (NKCC)1 (N16, sc-21545); the rabbit polyclonal anti-histamine H_1_ receptor (H300, sc-20633), anti-aquaporin (AQP)1 (H-56; sc-20810), anti-AQP2 (H-40; sc-28629), anti-AQP3 (H-80; sc-20811); mouse monoclonal anti-AQP7 (D-12; sc-376407) and histamine H_3_ receptor (D5, sc-390140) antibodies, as well as UltraCruz Autoradiography Film were from Santa Cruz Biotechnology (Dallas, TX, USA); the rabbit polyclonal anti-NHE3 (GTX41967; lot number 821700650) was from Gentex (San Antonio, TX, USA). The Alexa Fluor 594 AffiniPure bovine anti-goat and donkey anti-rabbit antibodies were from Jackson ImmunoResearch Laboratories, Inc. (West Grove, PA, USA). The mouse peroxidase-labeled and rabbit peroxidase-labeled secondary antibodies were from Cell Signaling Technology Inc. (Danvers, MA, USA). The BCA™ Protein Assay Kit was from Thermo Fisher Scientific (Waltham, MA, USA). The Opti-Protein XL Marker was from Applied Biological Materials Inc. (Richmond, BC, Canada) The Immobilon^®^ PVDF transfer membrane was from Merck Millipore (Milan, Italy). The Acrylamide/Bis solution 29:1 and the Albumin bovine modified Cohn Fraction V (BSA), pH 7.0, were from SERVA (Heidelberg, Germany). The WesternBright™ Quantum detection kit for the chemiluminescent detection and the Western Blot Strip-it Buffer were from Advansta (Menlo Park, CA, USA). Secondary mouse and rabbit biotinylated antibodies, Vectastain ABC Elite kit, and 3′,3-diaminobenzidine tetrahydrochloride were from DAB, Vector Laboratories, Inc. (Burlingame, CA, USA).

### 2.2. Animal Care and Ethics Statement

Twenty-six 5–13-week-old male C57BL6J (Charles River Laboratories, Calco, Italy) and 26 H_4_R^−/−^ mice backcrossed to CB57 background (generated by Lexicon Genetics, Woodlands Park, TX, USA, [9] and provided by Janssen Research & Development, LLC La Jolla, CA, USA) were maintained in compliance with the Italian regulations on the protection of animals used for experimental and other scientific purposes (D.M. 116/92) and the European Council directives (No. 2010/63/EU) and with the Principles of Laboratory Animal Care (NIH No. 85-23, revised 2011). The animals were kept at constant environmental and nutritional conditions at 25 ± 2 °C with alternating 12 h light and dark cycles and fed with a standard diet during a 1-week adaptation period. They were fed with a standard pellet diet (Piccioni, Settimo Milanese, Milan, Italy) and watered ad libitum. The scientific project was approved by the Ethical Committee of Turin University and the Italian Ministry of Health. The sample size was determined by applying the Fleiss test for an unmatched case-control study as power analysis. The confidence interval was 90%, the power was at 85%, and the alpha level was set at 0.05. This design provides the power to investigate the differences in renal function between the different groups.

### 2.3. Experiment Protocol

Diabetes was induced by a multiple low-dose streptozotocin (STZ)-intraperitoneal injection (50 mg/kg per day; STZ freshly made in 0.1 mol/L citrate buffer, pH 4.5) for 5 consecutive days, repeated after 8 weeks [19]. Control animals were treated with vehicles alone (n = 9 for each genotype). Animals matched for age between control and STZ were used. All the animals’ ages were represented in both groups. Diabetes was defined as fasting blood glucose level ≥200 mg/dL [20], and the onset of diabetes was evaluated by measuring 6 h fasting blood glucose using a Glucocard MX Blood Glucose Meter. Weight, food, and water intake were recorded weekly. At the end of the experimental period, mice were anaesthetized with isoflurane and sacrificed by cardiac exsanguination. Blood and kidneys were collected for biochemical and morphological analyses on renal function. 

### 2.4. Renal Function Evaluations

Twenty-four-hour urine collection was performed using the metabolic cage, and urine volume and pH were determined. Urinary protein excretion (UPE) was measured by Bradford method using Bovine Serum Albumin as the standard. Albuminuria was determined by ELISA. Creatinine was measured on both plasma and urine samples by a High-Performance Liquid Chromatography (HPLC) reverse-phase method as previously described [21,22].

### 2.5. Morphological Analysis

Kidney specimens were fixed by immersion in 4% paraformaldehyde, 0.1 M phosphate buffered saline (PBS) pH 7.4 overnight, embedded in paraffin. Therefore, the specimens were cut into 5 µm thick sections. Hematoxylin and eosin staining were carried out in order to analyze the gross tissue organization. Twenty microscopical fields/specimen were randomly selected and were digitalized at 200× and 400× using a digital camera connected to a light microscope (Olympus AX70). The glomerular injury was determined by measuring the glomerular area using ImageJ software (version 1.48v; National Institutes of Health, Bethesda, MD, USA) on 15 glomeruli from each mouse with hematoxylin and eosin staining (magnification: 400×) [23]. The number of injured tubules in 10 random images per cortical section was counted using 200× magnification with hematoxylin and eosin staining.

### 2.6. IL-6 Renal Content Assessment

IL-6 tissue content was determined on kidney lysate by the Mouse IL-6 Quantikine ELISA Kit, according to the manufacturer’s instructions. Total protein content spectrophotometrically measured by a micro-BCA™ Protein Assay Kit was used for normalization. 

### 2.7. Immunohistochemistry

Conventional immunohistochemical procedures were employed as described previously [7]. Briefly, immunoperoxidase staining for histamine H_1_, H_2_, and H_3_ receptors, NKCC1, AQP1, AQP2, AQP3, and AQP7 was performed on 5 μm sections for formalin-fixed tissue using sodium citrate 10 mM pH 6.9 as antigen retrieval. Sections were incubated overnight with anti-histamine H_1_, H_2_, and H_3_ receptors, -NKCC1, AQP-1, -AQP2, -AQP3, and -AQP7 (4 μg/mL) at 4 °C, followed by a three-layer streptavidin–biotin–peroxidase complex staining method. Tissue was also screened in the absence of the primary antibodies. In all cases, a significant part of the staining elicited by the untreated antibody was abolished. All sections were stained or immunostained in a single session to minimize artifactual differences in the staining. Photomicrographs of the histological slides were randomly taken with a digital camera connected to a light microscope equipped with a ×40 objective (Leica DM750 or Olympus AX70 for AQP7).

### 2.8. Immunofluorescence Analysis

Megalin and NHE3 immunoreactivity was determined on 5 µm thick tissue sections. The sections were deparaffinized and re-hydrated, followed by microwave antigen retrieval in 10 mM sodium citrate, pH 6.0. In order to quench the autofluorescence and minimize the non-specific binding, sections were incubated in 2 mg/mL glycine for 10 min and then for 20 min at room temperature with 1.5% bovine serum albumin in PBS pH 7.4. Sections were subsequently incubated overnight with goat polyclonal anti-megalin or rabbit polyclonal anti-NHE3. The immunoreactions were revealed by incubation with bovine anti-goat or donkey anti-rabbit Fluor 594-coniugated IgG antibody for 2 h at room temperature. Negative controls were carried out by omitting the primary antiserum. The immunoreaction products were observed, and pictures were acquired with Apotome systems (Zeiss). Data analysis and measurements were performed with ImageJ software.

### 2.9. Immunoblotting

Kidneys randomly selected from 5 animals/group were lysed in cold buffer (10 mM Tris/HCl pH 7.4, 10 mM NaCl, 1.5 mM MgCl_2_, 2 mM Na_2_ EDTA, 1% Triton X-100), supplemented with 10× Sigmafast Protease Inhibitor cocktail tablets. Total protein content was measured spectrophotometrically using a micro-BCA™ Protein Assay Kit. Forty micrograms of total proteins were randomly electrophoresed by SDS-PAGE and blotted onto PVDF membranes. The membranes were incubated overnight at 4 °C with rabbit polyclonal anti-NHE3, AQP1, AQP2, or mouse monoclonal anti-AQP7. The rabbit polyclonal anti-β-actin antibodies were used as a control. The immunoreactive bands were detected using rabbit or goat peroxidase-labeled secondary antibody and enhanced by WesternBright™ Quantum detection kit. Chemioluminescence signal was captured by X-ray film exposure. The densitometric analysis was performed by ImageJ software.

### 2.10. Statistical Analysis

Data were reported as mean values (± standard error of the means, S.E.M.). Statistical analysis was performed using a one-way analysis of variance (ANOVA). Post hoc calculations applying Tukey’s multiple comparisons test were made with Prism 9 statistical software (GraphPad Software, Inc., San Diego, CA, USA). Significance was set at a probability value (*p*) of <0.05.

## 3. Results

### 3.1. Comparison between Wild-Type and H_4_R^−/−^ Mice on Histamine Receptor Expression

**Figure 1:**
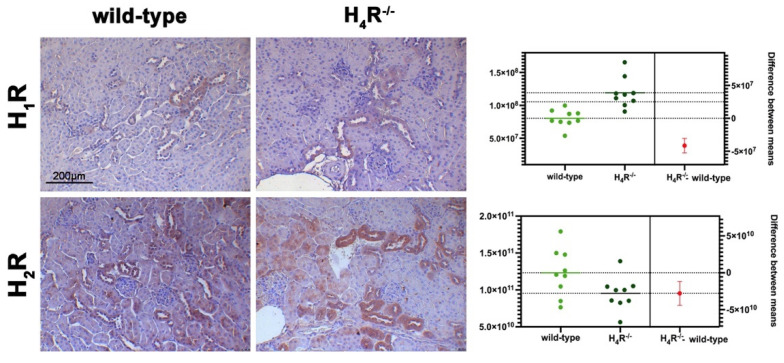
Comparison of histamine H_1_ and H_2_ receptor expression between wild-type and H_4_R^−/−^ mice. Micrographs represent the immunolabeling of transverse kidney sections with specific anti-histamine H_1_ and H_2_ antibodies (20× magnification). Positive staining area/total area was determined by color deconvolution, and the estimation plot of Welch’s *t*-test has been reported.

The expression of the histamine receptors in both wild-type and H_4_R^−/−^ mice was evaluated. As shown in Figure 1, only histamine H_1_ and H_2_ receptors were different between the two genotypes. Histamine H_1_ receptor was over-expressed in H_4_R^−/−^ mice. On the contrary, wild-type mice showed a higher level of the histamine H_2_ receptor.

### 3.2. Comparison between Wild-Type and H_4_R^−/−^ Mice on Functional Parameters

During the observation period of 116 days, wild-type and H_4_R^−/−^ animals showed similar body growth reaching, at the end of the experimental period, an average weight of 28.37 ± 1.68 and 29.57 ± 2.71 g, respectively, although the mean baseline weights were different at 21.52 ± 1.73 and 23.54 ± 2.33 g (*p* < 0.05).

The data reported in Table 1 on the renal functional parameters referred to the end of the experimental period (116 days). 

**Figure 2:**
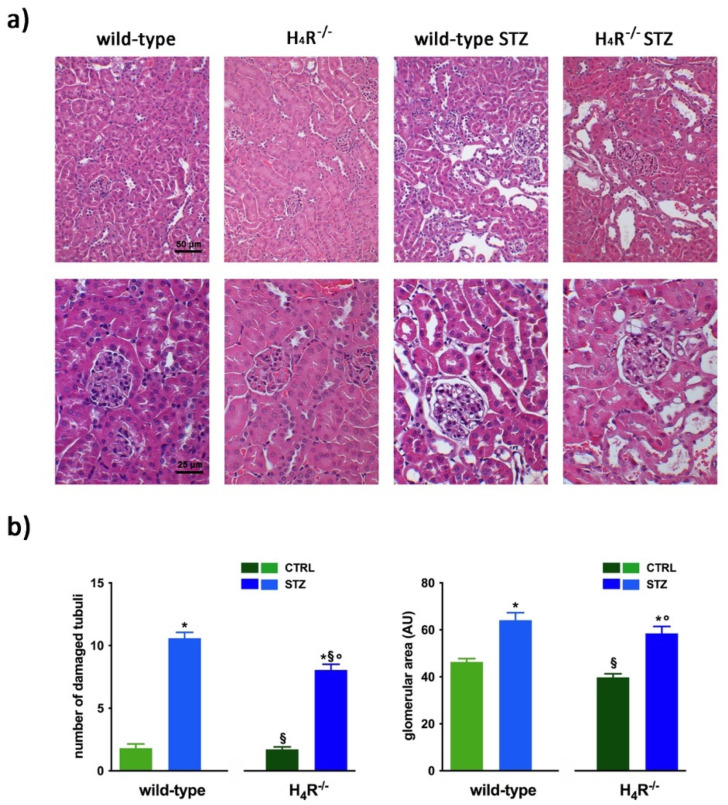
Comparison of gross tissue architecture between wild-type and H_4_R^−/−^ mice: (**a**) Representative micrographs showing hematoxylin and eosin staining at day 116 highlighting tubular and glomerular diabetic-associated alterations magnification: 200× in upper panels and 400× in lower panels; (**b**) Quantification of tubular damage and glomerular area. Data are expressed as the number of damaged tubules and glomerular area (Arbitrary Unit, AU), respectively. Data are expressed as the mean ± S.E.M.; * *p* < 0.05 vs. wild-type; ^§^ *p* < 0.05 vs. STZ wild-type; ° *p* < 0,05 STZ H_4_R^−/−^ vs. H_4_R^−/−^.

The comparison between the urine volumes collected after 24 h showed a significant difference between wild-type and H_4_R^−/−^ animals (1.33 ± 0.01 and 0.44 ± 0.10, respectively; *p* < 0.05). Similarly, UPE, albumin excretion, as well as the creatinine-to-albumin ratio (ACR) in the wild-type group were significantly higher than that of H_4_R^−/−^ (0.26 ± 0.04 vs. 0.09 ± 0.03 for albuminuria and 17.74 ± 1.63 vs. 8.67 ± 2.89 for ACR; *p* < 0.05). However, the urinary pH and creatinine clearance (CrCl) did not show any significant differences between the two groups (Table 1). Mice age did not affect the renal function changes observed. Despite the described differences in some urine parameters, the morphological analysis by hematoxylin and eosin staining revealed comparable renal architecture between wild-type and H_4_R^−/−^ mice (Figure 2a) without signs of inflammatory infiltration as well as tubular or glomerular damage (Figure 2a,b). 

**Figure 3:**
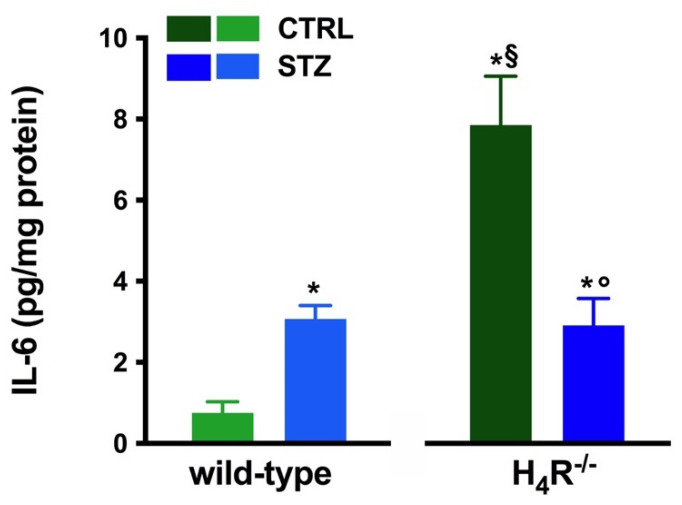
Comparison of IL-6 expression in kidney tissue between wild-type and H_4_R^−/−^ mice. Expression of IL-6, reported as pg/mg of tissue homogenate protein (i.e., pg/mg protein), was determined by the enzyme-linked immunosorbent assay (ELISA). Data are expressed as the mean (pg/mg protein) ± S.E.M.; * *p* < 0.05 vs. wild-type; ^§^ *p* < 0.05 vs. STZ wild-type; ° *p* < 0,05 STZ H_4_R^−/−^ vs. H_4_R^−/−^.

However, the analysis of the intrarenal inflammation marker IL-6 shown in Figure 3 demonstrates a higher level of renal IL-6 in H_4_R^−/−^ mice than the wild-type. 

### 3.3. Diabetic Nephropathy Development in H_4_R^−/−^ Mice

**Figure 4:**
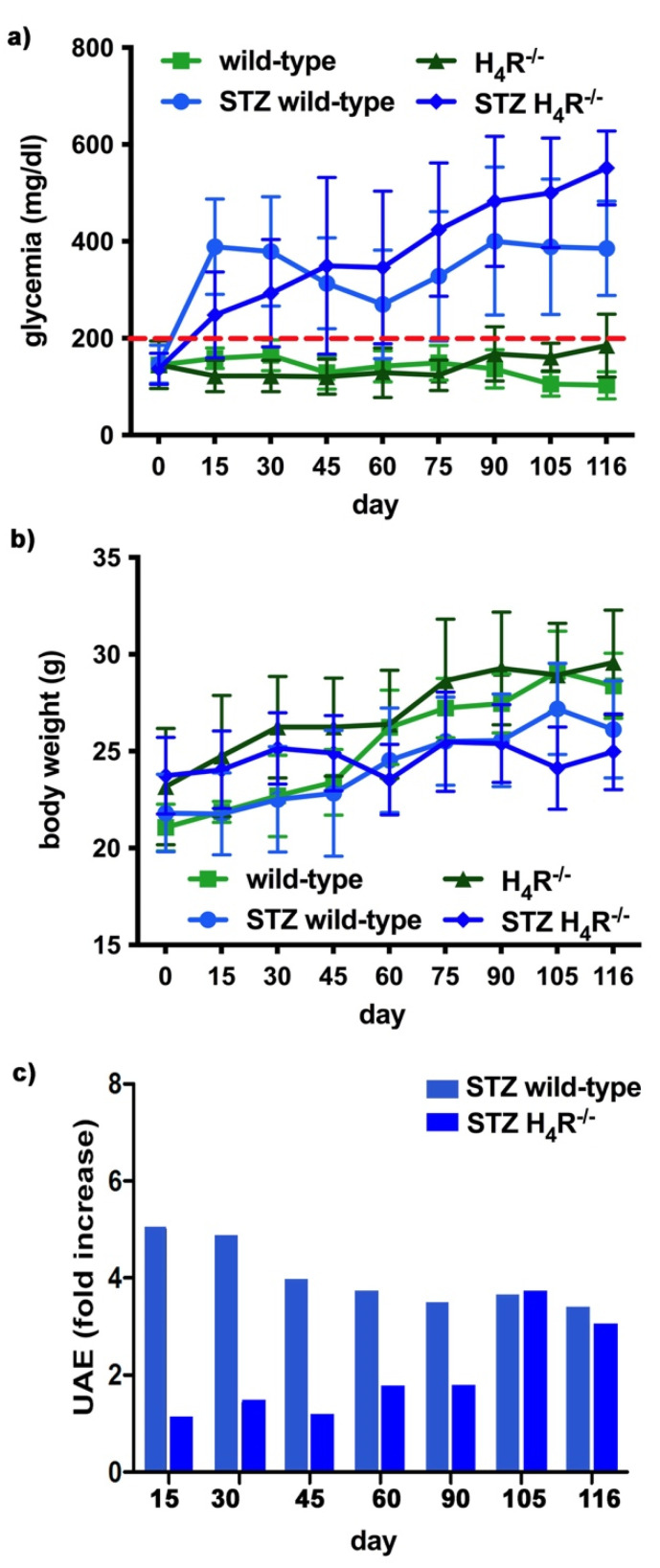
Comparison of diabetic wild-type and H_4_R^−/−^ mice: (**a**) Six-hour fasting blood glucose was recorded fortnightly using a Glucocard MX Blood Glucose Meter. Data are expressed as mean ± S.E.M. The red line identifies the 200 mg/dL fasting blood glucose level cut used to assess the diabetes onset; (**b**) Body weight was monitored and recorded constantly throughout the experimental period. Data are expressed as mean ± S.E.M.; (**c**) Albuminuria progression was monitored over time on urine samples collected over 24 h. The data are expressed as fold increases compared to the relatively healthy controls. UAE = albuminuria.

The basal glycemic level at day 116 was significantly higher in H_4_R^−/−^ animals compared to wild-type counterpart (184.77 ± 21, 67 mg/dL vs. 102.11 ± 20.03 mg/dL; *p* < 0.05), although lower than the cut-off of 200 mg/dL, defining diabetes [20] for both the genotypes. One week after the induction of diabetes, more than a half of the animals showed a glycemia ≥200 mg/dL; the glycemic level raised over 200 mg/dL within 14 days and remained severe [20] (300–600 mg/dL; *p* < 0.05 vs. control) through the observation period, irrespectively of the animal age in both wild-type and H_4_R^−/−^ mice (Figure 4a and Supplementary Materials Tables S3 and S4). The rise in glucose glycemic was paralleled by a reduction in body growth consistent with the type 1 diabetes model adopted: on day 116, the STZ wild-type group reached an average weight of 26.06 ± 2.44 g, and the STZ H_4_R^−/−^ 24.97 ± 1.95 g (Figure 4b and Supplementary Materials Tables S1 and S2). 

In hyperglycemic conditions, histopathological changes, the hallmark of diabetic nephropathy were present. In particular, glomeruli were hypertrophic and displayed an increase in the glomerular area irrespectively to the genotype, and tubular atrophy was evident, although H_4_R^−/−^ diabetic mice showed a lower number of damaged tubules compared to the wild-type counterpart (Figure 2b). Again, no evident signs of inflammatory infiltration were detectable in the different groups (Figure 2a). However, IL-6 was significantly increased in STZ wild-type animals compared to their control. On the contrary, the STZ H_4_R^−/−^ group showed downregulation of IL-6 with respect to H_4_R^−/−^ mice, reaching levels comparable to that of STZ wild-type animals (Figure 3). Looking at the renal function parameters in Table 1, diabetic animals showed values consistent with the renal damage characteristic of hyperglycemia: urine volume, urine acidity, UPE, albuminuria, ACR, and CrCl were significantly higher (*p* < 0.05) compared to the respective controls. However, the two genotypes present exciting differences. STZ wild-type animals showed lower polyuria (+8.70-fold) than the STZ H_4_R^−/−^ group, in which the fold increase in urinary volume compared to their healthy controls was +49.68. Similarly, proteinuria was increased by 5.70-fold in STZ wild-type animals and 28.74-fold in the STZ H_4_R^−/−^ group (*p* < 0.05). However, the increase in ACR was more significant in wild-type diabetics than in those of H_4_R^−/−^ (15.07 vs. 6.56; *p* < 0.05). Interestingly, the progression of albuminuria was different between the two genotypes. Indeed, the rise in albuminuria over time is slower in the STZ H_4_R^−/−^ group, reaching the fold increase registered in STZ wild-type animals only at day 105 (Figure 4c). Again, no significant difference was found for both urinary pH and CrCl (Table 1). The data reported and the differences found suggest that the H_4_ receptor is involved in tubular reabsorption phenomena.

### 3.4. Megalin Expression in Wild-Type and H_4_R^−/−^ Mice

**Figure 5:**
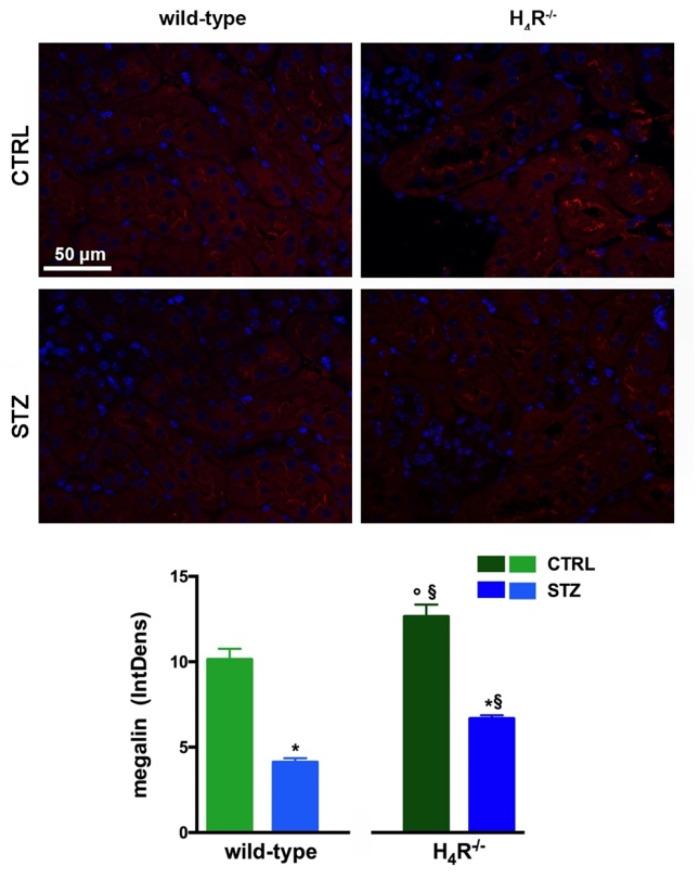
Comparison of megalin expression between wild-type and H_4_R^−/−^ mice. Micrographs at 40× magnification showing the immunofluorescence analysis of megalin (red). Nuclei were stained with DAPI (blue). Densitometric analysis of megalin expression was determined by immunofluorescence analysis. Expression levels over control are expressed as the mean (IntDen) ± S.E.M. (n = 10); * *p* < 0.05 vs. wild-type; ^§^ *p* < 0.05 vs. STZ wild-type; ° *p* < 0.05 STZ H_4_R^−/−^ vs. H_4_R^−/−^.

The data reported in Table 1 underline significant differences in albumin reabsorption between wild-type and H_4_R^−/−^ mice. Therefore, the expression of megalin, an essential endocytic receptor of the proximal tubular apparatus involved in the uptake of glomerular-filtered albumin [24], was evaluated. Consistent with the functional data, the immunofluorescence analysis of megalin revealed a significant increase in protein expression in the H_4_R^−/−^ group compared to the wild-type group (Figure 5). These differences in megalin basal expression were also found when diabetic mice were compared. Indeed, both wild-type and H_4_R^−/−^ mice showed a significant reduction of megalin, consistent with diabetic renal damage. However, the megalin loss was significantly reduced in H_4_R^−/−^ mice (Figure 5). These differences could explain, at least in part, the functional differences observed on the ACR. 

### 3.5. NHE3 Expression in Wild-Type and H_4_R^−/−^ Mice

**Figure 6:**
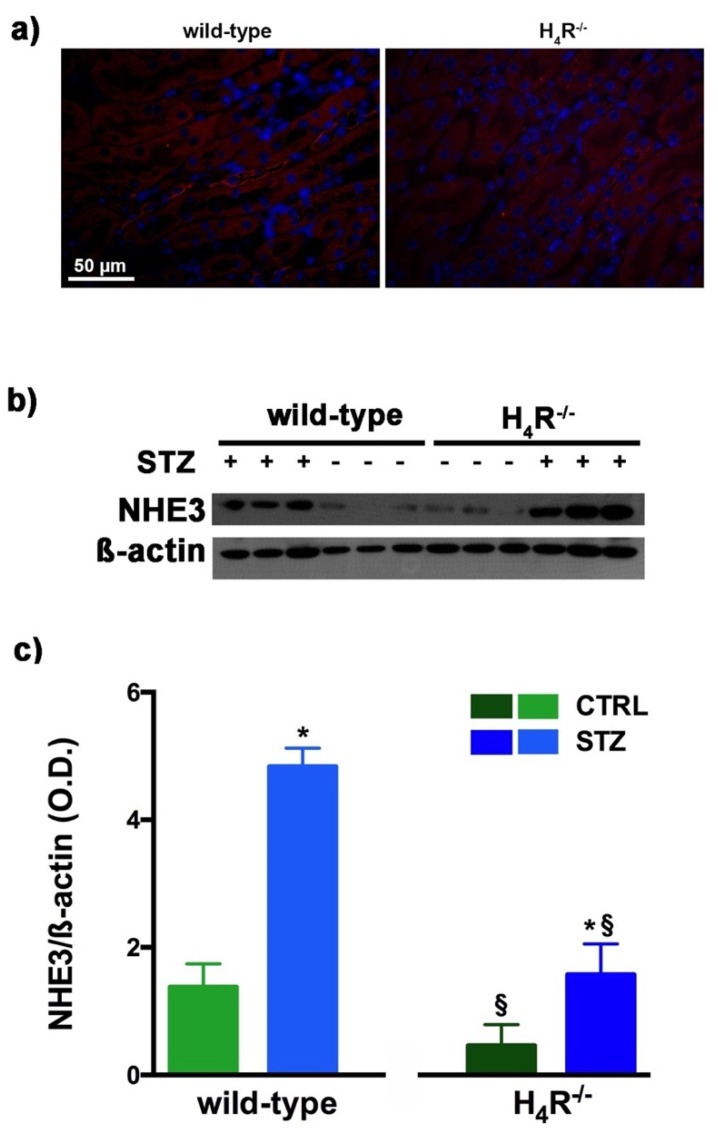
Comparison of NHE3 expression between wild-type and H_4_R^−/−^ mice. Micrographs at 40× magnification showing the immunofluorescence analysis of NHE3 (red). Nuclei were stained with DAPI (blue) (**a**); Representative radiograph of NHE3 in kidney tissue determined by immunoblotting (**b**); The densitometric analysis (**c**) was performed, and expression levels normalized to β-actin are expressed as the mean ± S.E.M. of 3 animals/group; * *p* < 0.05 vs. wild-type; ^§^ *p* < 0.05 vs. STZ wild-type.

As megalin expression is inversely correlated with that of NHE3 [25], we also evaluated the expression of this tubular exchanger. As reported in Figure 6a, control animals, both wild-type and H_4_R^−/−^ showed a weak fluorescence intensity, more localized in the apical area. The Western blot analysis revealed a single 95 kDa (molecular weight predicted for NHE3) species, demonstrating a significantly reduced expression of NHE3 in H_4_R^−/−^ mice. The exchanger was over-expressed in the diabetic groups but the basal differences between the two genotypes were retrieved in diabetic animals (Figure 6b,c). These data, therefore, further confirm the potential role of the H_4_ receptor in regulating tubular reabsorption. 

### 3.6. AQPs Expression in Wild-Type and H_4_R^−/−^ Mice

**Figure 7:**
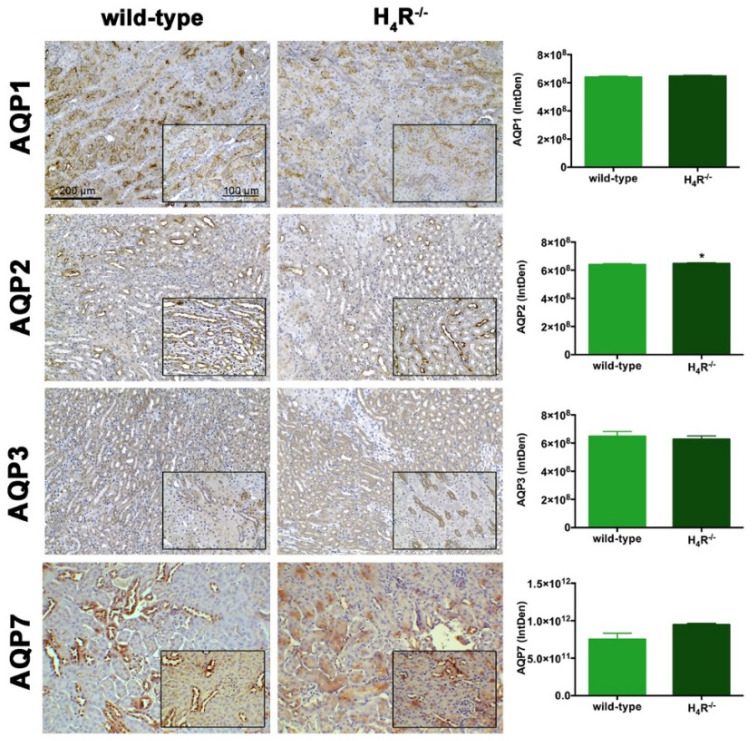
Comparison of AQPs expression between wild-type and H_4_R^−/−^ mice. Micrographs at 20× and 40× (insert) magnification of transverse kidney sections, immunolabeled with specific anti-AQP1, -AQP2, AQP3, and AQP7 antibodies. Positive staining area/total area was determined by color deconvolution. Results are the mean ± S.E.M. of the IntDen; * *p* < 0.05 vs. wild-type.

The urine volume differences observed between wild-type and H_4_R^−/−^ mice point out possible differences in the expression pattern of AQPs, a family of channel-forming transmembrane proteins differentially involved in water balance, included body water homeostasis [26]. Among them, AQP1, 3, and 7 are mainly expressed on the proximal tubular segment of the nephron. As shown in Figure 7, no significant differences were found. 

**Figure 8:**
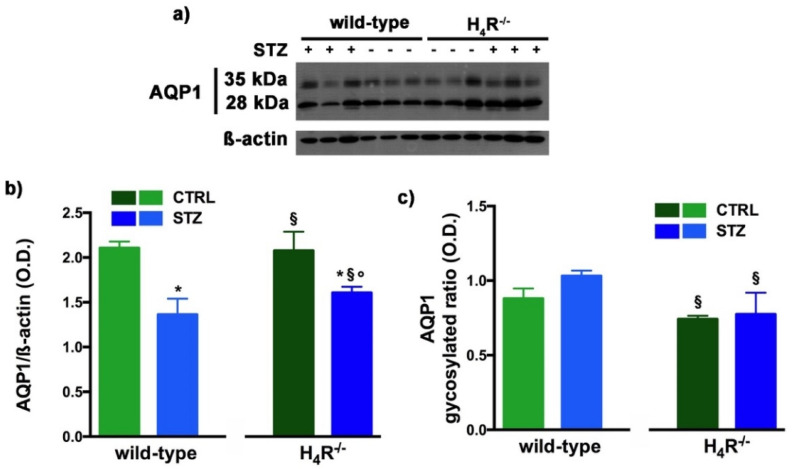
Comparison of AQP1 expression between wild-type and H_4_R^−/−^ mice. Representative radiograph of AQP1 in kidney tissue determined by immunoblotting (**a**); The densitometric analysis of the AQP1 overall content was performed, and expression levels, normalized to β-actin, are expressed as the mean ± S.E.M. of 3 animals/group (**b**); The glycosylation ratio was evaluated and was expressed as the mean ± S.E.M. of 3 animals/group (**c**); * *p* < 0.05 vs. wild-type; ^§^ *p* < 0,05 vs. STZ wild-type; ° *p* < 0,05 STZ H_4_R^−/−^ vs. H_4_R^−/−^.

**Figure 9:**
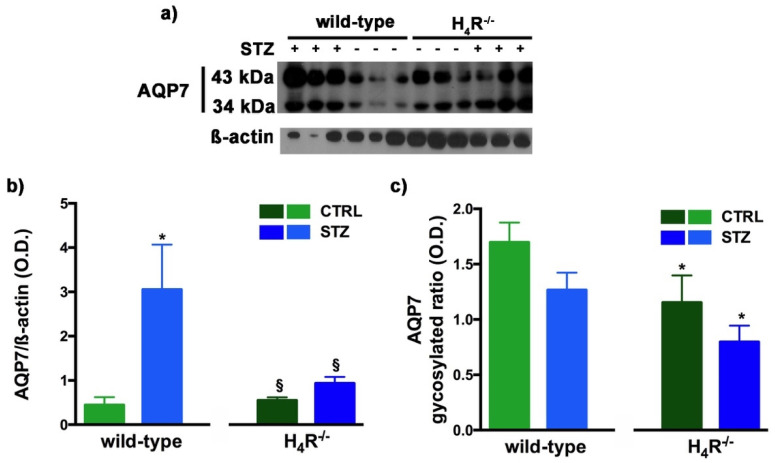
Comparison of AQP7 expression between wild-type and H_4_R^−/−^ mice. Representative radiograph of AQP7 in kidney tissue determined by immunoblotting (**a**); The densitometric analysis of the AQP7 overall content was performed, and expression levels, normalized to β-actin, are expressed as the mean ± S.E.M. of 3 animals/group (**b**); The glycosylation ratio was evaluated and was expressed as the mean ± S.E.M. of 3 animals/group (**c**); * *p* < 0.05 vs. wild-type; ^§^ *p* < 0,05 vs. STZ wild-type.

However, the Western blot analysis revealed exciting differences in the balance between the glycosylated and non-glycosylated forms of AQP1 and AQP7 (Figure 8 and Figure 9). 

As reported in Figure 8a, the Western blot analysis revealed for AQP1 two bands corresponding to the glycosylated (35 kDa) or the non-glycosylated (28 kDa) form [27]. The glycosylation ratio in H_4_R^−/−^ healthy or diabetic mice was lower than wild-type animals (Figure 8c). The overall content of AQP1 showed a significant reduction in diabetic animals compared to the respective controls. However, STZ wild-type animals showed significantly lower expression of AQP1 (Figure 8b) but a higher glycosylation ratio (Figure 8c) than STZ H_4_R^−/−^. 

A similar glycosylation ratio analysis was also performed for AQP7. Two bands for AQP7 were revealed (Figure 9a): one with a higher molecular weight (43 kDa) and one with a lower molecular weight (34 kDa), which correspond respectively to the glycosylated and non-glycosylated form [28]. The glycosylation ratio in H_4_R^−/−^ mice was lower than wild-type animals (Figure 9c). Diabetic wild-type mice but not H_4_R^−/−^ showed an increase in AQP7 (Figure 9b) without significant differences in the glycosylation ratio compared to the relative controls (Figure 9c). 

**Figure 10:**
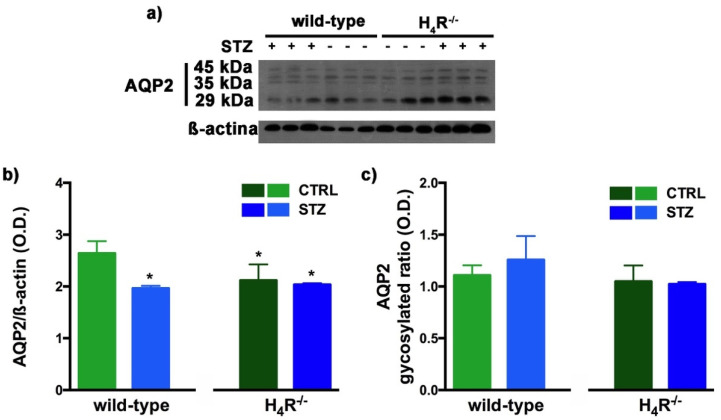
Comparison of AQP2 expression between wild-type and H_4_R^−/−^ mice. Representative radiograph of AQP2 in kidney tissue determined by immunoblotting (**a**); The densitometric analysis of the AQP2 overall content was performed, and expression levels normalized to β-actin are expressed as the mean ± S.E.M. of 3 animals/group (**b**); The glycosylation ratio was evaluated and was expressed as the mean ± S.E.M. of 3 animals/group (**c**); * *p* < 0.05 vs. wild-type.

Finally, AQP2 expression was evaluated. This AQP, located on the apical cell membranes of the kidney’s collecting duct principal cells, is vasopressin-sensitive, and is the most well-studied AQP in the kidney [29]. Notably, the expression of AQP2 was lower in the H_4_R^−/−^ (Figure 7 and Figure 10). 

The Western blot analysis of AQP2 again detected a multi-band profile with one band at about 45 kDa, two at about 35 kDa, and the last at 29 kDa (Figure 10a). This spectrum is again compatible with AQP2 glycosylation, which determines its localization at the level of the apical membrane of the main cells of the collecting duct [30]. As shown in Figure 10b, a significant reduction in the expression of AQP2 was observed not only in H_4_R^−/−^ mice but also in both diabetic wild-type and H_4_R^−/−^ animals. However, no differences in the glycosylation ratio were registered (Figure 10c). The AQP pattern evaluation data collectively suggest that histamine could regulate the renal water balance through the H_4_ receptor.

### 3.7. NKCC1 Expression in Wild-Type and H_4_R^−/−^ Mice

**Figure 11:**
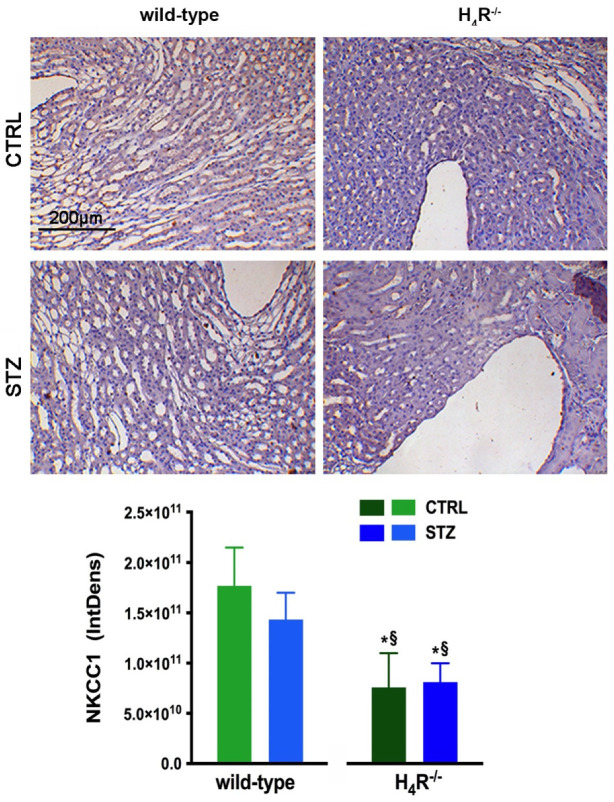
Comparison of NKCC1 expression between wild-type and H_4_R^−/−^ mice. Micrographs at 20× magnification of transverse kidney sections, immunolabeled with specific anti-NKCC1 antibody. Densitometric analysis of NKCC1 expression was determined by color deconvolution analysis. Expression levels over control are expressed as the mean (IntDen) ± S.E.M. (n = 10); * *p* < 0.05 vs. wild-type; ^§^ *p* < 0.05 vs. STZ wild-type.

With NKCC1, being a key cotransporter in the kidney, we also evaluated its expression. More precisely, we investigated the expression of the NKCC1 cotransporter, the form that is widely distributed throughout the body but especially abundant in the kidney. Like AQP-2, NKCC1 is expressed on the outer medullary collecting duct but on the α-intercalated cells [31]. As shown in Figure 11, the immunohistochemistry analysis revealed a reduced basal expression of NKCC1 in H_4_R^−/−^ animals compared to their wild-type counterpart.

## 4. Discussion

In conclusion, the results obtained support the hypothesis that the H_4_ receptor is involved in tubular reabsorption processes. Indeed, compared to their wild-type counterpart, at basal, H_4_R^−/−^ mice showed significant alterations in renal function with a reduction of both urinary volume and excretion of proteins, albumin in particular. The urine of the H_4_R^−/−^ mice were also less concentrated than wild-type. These events find a possible explanation in the constitutive over-expression of megalin and parallel down-regulation of NHE3 and NKCC1 proteins in the H_4_R^−/−^ mice. The presence of megalin at the level of the apical membrane of the proximal tubule is positively correlated with its ability to reabsorb albumin and the proteins present in the glomerular filtrate [32]. The mechanism by which histamine could modulate megalin expression is still unknown. However, it is possible to speculate that similarities exist between the histamine H_4_ receptor and the angiotensin II receptor AT1. The activation of the AT1 receptor, a Gi-coupled receptor like the histamine H_4_ receptor, suppresses the megalin expression [33]. Therefore, suppressing the histamine H_4_ receptor could be responsible for an imbalance of cAMP signaling, accounting for increased megalin basal levels. However, a possible contribution of other histamine receptors, particularly histamine H_1_ receptor over-expression, could not be ruled out. Indeed, in our study, a compensation for histamine receptor subtypes occurs in the kidney of H_4_R^−/−^ mice: the histamine H_1_ receptor was over-expressed, and the histamine H_2_ receptor was down-regulated. On the contrary, the basal expression of histamine H_3_ receptor remains scantly limited to some restricted areas of the collecting ducts [34]. 

Conversely, NHE3 is less expressed in H_4_R^−/−^ mice. NHE3 is considered the central mediator of sodium uptake in the proximal tubule and is reported to regulate urinary pH. However, parallel to the reduction in NHE3, only a trend of increase in pH was observed in H_4_R^−/−^ mice. However, the difference in pH was not statistically significant, but this may be due to the system used for the assessment. The test strips are a semi-quantitative method and are therefore not very sensitive to the slight variation observed (+0.15-fold).

It should be noted that the NHE3 exchanger shares the anatomical location and signal pathways with the histamine H_4_ receptor. Both histamine H_4_ receptor and NHE3 are present at the level of the proximal tubule and of the ascending tract, often of the loop of Henlé [7,35,36]. Moreover, a cross-talk between the H_4_ receptor and NHE3 could occur through cAMP. The H_4_ receptor is coupled to the Gi protein, and its activation in proximal tubular cells involves an intracellular reduction of cAMP [37] that could reduce NHE3 expression. Indeed, cAMP inhibits the expression of NHE3 [38,39]. However, the cross-talk could occur at NHE3 regulation by vasopressin, which causes its inhibition in the ascending tract of the loop of Henlé [40]. Starting from the consideration that the knockout mice used are total body knockout, the deletion of the H_4_ receptor may have an effect on the production and release of vasopressin by the posterior pituitary and hypothalamic neurons, where all four receptors for histamine are present [6]. Therefore, the deletion of the H_4_ receptor may affect the production and release of vasopressin by the posterior pituitary and hypothalamic neurons. Consistently, systemic histamine infusion causes oliguria following an increase in vasopressin release [41] and reducing urinary volume in diabetic mice treated with the H_4_ antagonist JNJ39758979 [8]. The H_4_ receptor could therefore participate in the regulation of vasopressin release, and its deletion/inhibition could enhance the release of vasopressin by histamine with a consequent antidiuretic effect. However, other receptors, such as the H_3_ receptor present both centrally [6] and on the apical membrane of the main cells of the collecting duct [34] could be involved. Finally, the role of histamine H_1_ and H_2_ receptors in regulating renal hemodynamics cannot be ruled out [18]. 

More complex is the interpretation of the effect of AQPs. AQP1, AQP3, and AQP7 showed no difference between wild-type and H_4_R^−/−^ mice. On the contrary, AQP2 was down-regulated in H_4_R^−/−^ mice. Compared to other AQPs, limited information is available on the role of AQP7 in renal pathophysiology. However, the deletion of AQP7 alone has been associated with glyceroluria and only mild polyuria [42]. The reduction of AQP2 expression in H_4_R^−/−^ mice is consistent with the observed lower urine output. However, this appears to be an indirect effect of histamine H_4_ receptor deletion. Indeed, AQP2 is located on the collecting duct, where the histamine H_3_ receptor is expressed. Moreover, AQP2 is vasopressin sensitive. In particular, vasopressin has been reported to regulate AQP2 expression [43]. Therefore, central regulation of vasopressin release and/or histamine H_3_ receptor could account for that effect. The central regulation of vasopressin could account also for the effect observed on NKCC1 expression. Indeed, this cotransporter is directly and indirectly regulated by vasopressin [44]. Nevertheless, NKCC1 is critical to the release and/or action of vasopressin, renin and aldosterone, and the regulation of renal Na^+^, K^+^, Cl^−^, and water excretion [45]. 

The study’s second aim was to evaluate the propensity of H_4_R^−/−^ mice to develop diabetic nephropathy in an STZ-induced diabetes model. This model of renal damage was selected on the basis of the increase in renal levels of histamine already reported [18,46]. Interestingly, H_4_R^−/−^ mice displayed a higher basal glycemic state. After diabetes induction, H_4_R^−/−^ animals tended to develop severe progressive hyperglycemia with double blood glucose values at the end of the treatment period compared to the wild-type counterpart. This phenomenon could be explained by the role of histamine H_4_ in the immune response [47,48]. However, further studies are needed to clarify the mechanisms underlying the development of hyperglycemia. The use of an H_4_ antagonist, JNJ39758979, had not shown any effect on blood sugar [8]. In contrast, it was reported that the activation of histamine H_4_ receptors located at the spinal cord exerts a modulatory effect on blood glucose regulation [49,50]. However, the increased sensitivity to hyperglycemia may be due to the use of total body knockout mice instead of the conditional knockout with a selective tubular deletion, according to the renal histamine H_4_ receptor tubular localization [18]. Therefore, other histaminergic receptors may play a dominant role in this effect in H_4_R^−/−^ mice, possibly as a compensatory response. In addition, the absence of the H_4_R^−/−^ gene could alter the immuno-mediated response of pancreatic ß-cells elicited by the multiple low doses of STZ protocol [19]. Moreover, the proximal tubule is also deputed to glucose reabsorption through specific transporters such as SGLT2 [51], the target of anti-diabetic drugs known as glifozins [52]. As our data indicate Histamine H_4_ receptor as a modulator of tubular reabsorptive processes mediated by megalin, NHE3, and AQPs, it is possible to speculate that all the reabsorptive machinery of the tubules is involved, SGLT2 pathway included. 

The results obtained on renal functional parameters confirm, at least in part, the role of histamine H_4_ receptors in tubular reabsorption. Indeed, diabetic H_4_R^−/−^ animals showed lower albuminuria and lower ACR, although to a lower extent than what was observed previously with the histamine H_4_ receptor antagonist JNJ39758979 [8]. In parallel, the H_4_R^−/−^ mice showed higher megalin expression and a smaller increase in NHE3 than their counterpart. However, the overall assessment of renal function does not show a clear improvement in the knockout diabetic animal compared to the wild-type animal. Both urinary pH and CrCl do not show significant differences, and CrCl, in particular, suggests a significant increase in glomerular filtration. However, differences in glycemic levels between wild-type and knockout animals make the resulting kidney damage challenging to compare. Indeed, it is known that hyperglycemia is correlated in a dose- and time-dependent manner with the progression of diabetes-induced kidney damage [53]. Consistently, the albuminuria progression analysis showed that its onset was slower in diabetic H_4_R^−/−^ animals. Therefore, on this basis, it is possible to speculate that although the diabetic H_4_R^−/−^ animals did not show an improved renal function, the progression of damage may be delayed in terms of onset. The delayed progression of renal damage, confirmed by the number of damaged tubules, could contribute to the renal production of IL-6. This well-known marker of renal damage is increased at the basal level in H_4_R^−/−^ mice. This data is in keeping with the observed over-expression of the histamine H_1_ receptor. Indeed, it has been clearly reported that IL-6 secretion is fundamentally based on the histamine H_1_ receptor [54,55,56,57,58], more than the histamine H_4_ receptor [59]. However, the increased basal level in IL-6 does not correlate with any sign of inflammation. Actually, IL-6 elicits pro-inflammatory properties via the trans-signaling pathway and anti-inflammatory properties through the classic signaling pathway [60]. Unexpectedly, not only the basal level of IL-6 was higher in H_4_R^−/−^ mice, but also diabetic H_4_R^−/−^ mice showed lower IL-6 levels compared to their healthy control. However, this down-regulation of IL-6 in diabetic knockout animals could correlate with the slower progression of renal damage. 

The lack of effect on CrCl could also be explained by the purely tubular localization of the H_4_ receptor being absent at the glomerular level [18]. Consistently, in vitro [36] and in vivo [61,62] data suggested that among the histamine receptors, the H_1_ receptor is the one directly involved in hyperfiltration.

Furthermore, the urinary volume was unexpectedly higher in diabetic H_4_R^−/−^ animals, which showed a +49% increase in water excretion than the 8% increase measured for wild-type diabetics. 

This effect and the lack of effects on CrCl and extent of ACR seem to be not consistent with our previous findings on the renal protective effect of the Histamine H_4_ receptor antagonist JNJ39758979. Moreover, there were clear signs of hypocellularity and pro-inflammatory cell infiltration [8]. However, it has to be noticed that the studies used different animal strains: DBA2/J [8,62] in the previous, and C57BL6J in this new study. The two strains have different susceptibility to diabetic nephropathy and to STZ, and differ in some renal pathological changes [63,64]. Consistently, the severity of diabetes differed in the two studies, with animals in this last one displaying more severe hyperglycemia for a prolonged time, especially in the H_4_R^−/−^ diabetic group. Therefore, it is challenging to compare the entity of the effect evoked by JNJ39758979 in DBA2/J mice [8] or by the histamine H_4_ receptor deletion. 

The effect on the urinary volume could be due to an imbalance in histamine receptors due to the histamine H_4_ receptor deletion. We previously demonstrated that the histamine H_3_ receptor is expressed on the apical membrane of collecting ducts [34]. The anatomical distribution of this latter receptor is suggestive of its possible involvement in regulating water excretion. Consistently, our data demonstrated that in diabetic rats displaying a polyuric phenotype, the histamine H_3_ receptor is upregulated [34].

Moreover, data presented by our group at the 45th EHRS Meeting in Florence (11–14 May 2016) showed a positive correlation between histamine H_3_ receptor and urinary volume [9]. So far, it is possible that the histamine H_3_ receptor, no more balanced by the histamine H_4_ receptor, could account for the difference in the urine volume. However, the polyuric effect could be attributed to the expression of AQP1 and AQP7, whose full expression and membrane localization are significantly reduced in diabetic knockout animals. Indeed, double KO mice for AQP1/AQP7 exhibit reduced concentration, urine osmolarity, and increased urinary volume [65].

## 5. Conclusions

In conclusion, the use of H_4_R^−/−^ mice further support the hypothesis that the H_4_ receptor is involved in tubular reabsorption processes with particular regards to albumin uptake.

## Figures and Tables

**Table 1:** Renal function parameters at day 116.

	Wild-Type	H_4_R^−/−^	STZ Wild-Type	STZ H_4_R^−/−^
Urine volume (mL)	1.33 ± 0.01	0.44 ± 0.10 *	11.57 ± 0.91 *	21.86 ± 1.84 °^§^
pH ^#^	6.35 ± 0.16	6.5 ± 0.00	5.24 ± 0.03 *	5.37 ± 0.09 °
UPE (mg/24 h)	3.85 ± 0.11	1.1 ± 0.22 *	21.95 ± 1.63 *	31.62 ± 2.94 °^§^
Albumin excretion (µg/24 h)	0.26 ± 0.04	0.09 ± 0.03 *	0.91 ± 0.04 *	0.31 ± 0.06 °^§^
ACR (µg/mg)	17.74 ± 1.63	8.67 ± 2.89 *	267.38 ± 22.78 *	56.92 ± 6.89 °^§^
CrCl (mL/min)	0.49 ± 0.12	0.28 ± 0.11	1.66 ± 0.19 *	1.17 ± 0.75

# semi-quantitative analysis by dip-stick; UPE = urinary protein excretion; ACR = albumin-to-creatinine ratio; CrCl = creatinine clearance data are the mean ± S.E.M.; * *p* < 0.05 vs. wild-type; ^§^ *p* < 0.05 vs. STZ wild-type; ° *p* < 0.05 STZ H_4_R^−/−^ vs. H_4_R^−/−^.

## Data Availability

The data presented in this study are available in Tables S1–S4. Further row data are available on request from the corresponding author, A.C.R.

## Supplementary Materials

The following are available online at https://www.mdpi.com/article/10.3390/biom11101517/s1, Table S1: Row in vivo data: H_4_R^−/−^ mice weight, Table S2: Row in vivo data: wild-type mice weight, Table S3: Row in vivo data: H_4_R^−/−^ mice glycemia, Table S4: Row in vivo data: wild-type mice glycemia.

## References

[1] Nakamura T., Itadani H., Hidaka Y., Ohta M., Tanaka K. (2000). Molecular cloning and characterization of a new human histamine receptor, HH_4_R. Biochem. Biophys. Res. Commun..

[2] Oda T., Morikawa N., Saito Y., Masuho Y., Matsumoto S.-I. (2000). Molecular cloning and Characterization of a Novel Type of Histamine Receptor Preferentially Expressed in Leukocytes. J. Biol. Chem..

[3] Liu C., Ma X.-J., Jiang X., Wilson S.J., Hofstra C.L., Blevitt J., Pyati J., Li X., Chai W., Carruthers N. (2001). Cloning and pharmacological Characterization of a Fourth Histamine Receptor (H4) Expressed in Bone Marrow. Mol. Pharmacol..

[4] Seifert R., Strasser A., Schneider E.H., Neumann D., Dove S., Buschauer A. (2013). Molecular and cellular analysis of human histamine receptor subtypes. Trends Pharmacol. Sci..

[5] Neumann D., Schneider E.H., Seifert R. (2014). Analysis of histamine Receptor Knockout Mice in Models of Inflammation. J. Pharmacol. Exp. Ther..

[6] Panula P., Chazot P.L., Cowart M., Gutzmer R., Leurs R., Liu W.L.S., Stark H., Thurmond R.L., Haas H.L. (2015). International Union of Basic and Clinical Pharmacology. XCVIII. Histamine Receptors. Pharmacol. Rev..

[7] Rosa A.C., Grange C., Pini A., Katebe M.A., Benetti E., Collino M., Miglio G., Bani D., Camussi G., Chazot P.L. (2013). Overexpression of Histamine H_4_ receptors in the kidney of diabetic rat. Inflamm. Res..

[8] Pini A., Grange C., Veglia E., Argenziano M., Cavalli R., Guasti D., Calosi L., Ghè C., Solarino R., Thurmond R.L. (2018). Histamine H_4_ receptor antagonism prevents the progression of diabetic nephropathy in male DBA2/J mice. Pharmacol. Res..

[9] Hofstra C.L., Desai P.J., Thurmond R.L., Fung-Leung W.-P. (2003). Histamine H_4_ Receptor mediates Chemotaxis and Calcium Mobilization of Mast Cells. J. Pharmacol. Exp. Ther..

[10] Dunford P.J., O’Donnell N., Riley J.P., Williams K.N., Karlsson L., Thurmond R.L. (2006). The Histamine H_4_ receptor Mediates Allergic Airway Inflammation by Regulating the Activation of CD4+ T Cells. J. Immunol..

[11] Dunford P.J., Williams K.N., Desai P.J., Karlsson L., McQueen D., Thurmond R. (2007). Histamine H_4_ receptor antagonists are superior to traditional antihistamines in the attenuation of experimental pruritus. J. Allergy Clin. Immunol..

[12] Hartwig C., Munder A., Glage S., Wedekind D., Schenk H., Seifert R., Neumann D. (2014). The histamine H_4_-receptor (H_4_R) regulates eosinophilic inflammation in ovalbumin-induced experimental allergic asthma in mice. Eur. J. Immunol..

[13] Rossbach K., Schaper K., Kloth C., Gutzmer R., Werfel T., Kietzmann M., Bäumer W. (2015). Histamine H_4_ receptor knockout mice display reduced inflammation in a chronic model of atopic dermatitis. Allergy.

[14] Schirmer B., Bringmann L., Seifert R., Neumann D. (2018). In vivo evidence for Partial Activation of Eosinophils via the Histamine H4-Receptor: Adoptive Transfer Experiments Using Eosinophils from H_4_R−/− and H_4_R+/+ Mice. Front. Immunol..

[15] Sanna M.D., Ghelardini C., Thurmond R., Masini E., Galeotti N. (2017). Behavioural phenotype of Histamine H_4_ receptor knockout mice: Focus on central neuronal functions. Neuropharmacology.

[16] Sterle H., Nicoud M.B., Massari N.A., Delgado M.A.T., Ducloux M.V.H., Cremaschi G.A., Medina V.A. (2018). Immunomodulatory role of Histamine H_4_ receptor in breast cancer. Br. J. Cancer.

[17] Pini A., Obara I., Battell E., Chazot P.L., Rosa A.C. (2016). Histamine in diabetes: Is it time to reconsider?. Pharmacol. Res..

[18] Grange C., Gurrieri M., Verta R., Fantozzi R., Pini A., Rosa A.C. (2020). Histamine in the kidneys: What is its role in renal pathophysiology?. Br. J. Pharmacol..

[19] McEvoy R.C., Andersson J., Sandler S., Hellerström C. (1984). Multiple low-dose streptozotocin-induced diabetes in the mouse. Evidence for stimulation of a cytotoxic cellular immune response against an insulin-producing beta cell line. J. Clin. Investig..

[20] Chow B.S., Allen T.J. (2015). Mouse models for Studying Diabetic Nephropathy. Curr. Protoc. Mouse Biol..

[21] Dunn S.R., Qi Z., Bottinger E.P., Breyer M.D., Sharma K. (2004). Utility of endogenous creatinine clearance as a measure of renal function in mice. Kidney Int..

[22] Yuen P.S.T., Dunn S.R., Miyaji T., Yasuda H., Sharma K., Star R.A. (2004). A simplified method for HPLC determination of creatinine in mouse serum. Am. J. Physiol. Physiol..

[23] Grange C., Tritta S., Tapparo M., Cedrino M., Tetta C., Camussi G., Brizzi M.F. (2019). Stem cell-derived extracellular vesicles inhibit and revert fibrosis progression in a mouse model of diabetic nephropathy. Sci. Rep..

[24] De S., Kuwahara S., Saito A. (2014). The endocytic Receptor Megalin and its Associated Proteins in Proximal Tubule Epithelial Cells. Membranes.

[25] Girardi A., Di Sole F. (2012). Deciphering the mechanisms of the Na+/H+ exchanger-3 regulation in organ dysfunction. Am. J. Physiol. Physiol..

[26] Azad A.K., Raihan T., Ahmed J., Hakim A., Emon T.H., Chowdhury P.A. (2021). Human aquaporins: Functional diversity and Potential Roles in Infectious and Non-infectious Diseases. Front. Genet..

[27] Maunsbach A.B., Marples D., Chin E., Ning G., Bondy C., Agre P., Nielsen S. (1997). Aquaporin-1 water channel expression in human kidney. J. Am. Soc. Nephrol..

[28] Chen X.-F., Li C.-F., Lu L., Mei Z.-C. (2016). Expression and clinical significance of aquaglyceroporins in human hepatocellular carcinoma. Mol. Med. Rep..

[29] Su W., Cao R., Zhang X., Guan Y. (2020). Aquaporins in the kidney: Physiology and pathophysiology. Am. J. Physiol. Physiol..

[30] Hendriks G., Koudijs M., van Balkom B.W.M., Oorschot V., Klumperman J., Deen P.M.T., van der Sluijs P. (2004). Glycosylation is Important for Cell Surface Expression of the Water Channel Aquaporin-2 but Is Not Essential for Tetramerization in the Endoplasmic Reticulum. J. Biol. Chem..

[31] Wall S.M., Fischer M.P. (2002). Contribution of the Na^+^-K^+^-2Cl^−^ Cotransporter (NKCC1) to transepithelial Transport of H^+^, NH_4_^+^, K^+^, and Na^+^ in Rat Outer Medullary Collecting Duct. J. Am. Soc. Nephrol..

[32] Sun J., Hultenby K., Axelsson J., Nordström J., He B., Wernerson A., Lindström K. (2017). Proximal tubular Expression Patterns of Megalin and Cubilin in Proteinuric Nephropathies. Kidney Int. Rep..

[33] Hosojima M., Sato H., Yamamoto K., Kaseda R., Soma T., Kobayashi A., Suzuki A., Kabasawa H., Takeyama A., Ikuyama K. (2009). Regulation of megalin Expression in Cultured Proximal Tubule Cells by Angiotensin II Type 1A Receptor- and Insulin-Mediated Signaling Cross Talk. Endocrinology.

[34] Pini A., Chazot P.L., Veglia E., Moggio A., Rosa A.C. (2015). H3 receptor renal expression in normal and diabetic rats. Inflamm. Res..

[35] Vallon V., Schwark J.-R., Richter K., Hropot M. (2000). Role of Na+/H+ exchanger NHE3 in nephron function: Micropuncture studies with S3226, an inhibitor of NHE3. Am. J. Physiol. Physiol..

[36] Veglia E., Pini A., Moggio A., Grange C., Premoselli F., Miglio G., Tiligada K., Fantozzi R., Chazot P.L., Rosa A.C. (2016). Histamine type 1-receptor activation by low dose of histamine undermines human glomerular slit diaphragm integrity. Pharmacol. Res..

[37] Veglia E., Grange C., Pini A., Moggio A., Lanzi C., Camussi G., Chazot P.L., Rosa A.C. (2015). Histamine receptor expression in human renal tubules: A comparative pharmacological evaluation. Inflamm. Res..

[38] Yun C.H.C., Oh S., Zizak M., Steplock D., Tsao S., Tse C.-M., Weinman E.J., Donowitz M. (1997). cAMP-mediated inhibition of the epithelial brush border Na+/H+ exchanger, NHE3, requires an associated regulatory protein. Proc. Natl. Acad. Sci. USA.

[39] Honegger K.J., Capuano P., Winter C., Bacic D., Stange G., Wagner C.A., Biber J., Murer H., Hernando N. (2006). Regulation of sodium-proton exchanger isoform 3 (NHE3) by PKA and exchange protein directly activated by cAMP (EPAC). Proc. Natl. Acad. Sci. USA.

[40] Gunaratne R., Braucht D.W.W., Rinschen M.M., Chou C.-L., Hoffert J.D., Pisitkun T., Knepper M.A. (2010). Quantitative phosphoproteomic analysis reveals cAMP/vasopressin-dependent signaling pathways in native renal thick ascending limb cells. Proc. Natl. Acad. Sci. USA.

[41] Cacabelos R., Yamatodani A., Niigawa H., Hariguchi S., Nishimura T., Wada H. (1987). Histaminergic Neuromodulation of the Release of Vasopressin. Neuroendocrinology.

[42] Sohara E., Rai T., Sasaki S., Uchida S. (2006). Physiological roles of AQP7 in the kidney: Lessons from AQP7 knockout mice. Biochim. Biophys. Acta (BBA) Biomembr..

[43] Wilson J.L.L., Miranda C.A., Knepper M.A. (2013). Vasopressin and the regulation of aquaporin-2. Clin. Exp. Nephrol..

[44] Wakamatsu S., Nonoguchi H., Ikebe M., Machida K., Izumi Y., Memetimin H., Nakayama Y., Nakanishi T., Kohda Y., Tomita K. (2009). Vasopressin and hyperosmolality regulate NKCC1 expression in rat OMCD. Hypertens. Res..

[45] Wall S.M., Knepper M.A., Hassell K.A., Fischer M.P., Shodeinde A., Shin W., Pham T.D., Meyer J.W., Lorenz J.N., Beierwaltes W.H. (2006). Hypotension in NKCC1 null mice: Role of the kidneys. Am. J. Physiol. Physiol..

[46] Pini A., Verta R., Grange C., Gurrieri M., Rosa A.C. (2019). Histamine and diabetic nephropathy: An up-to-date overview. Clin. Sci..

[47] O’Mahony L., Akdis M., Akdis C.A. (2011). Regulation of the immune response and inflammation by histamine and histamine receptors. J. Allergy Clin. Immunol..

[48] Branco A.C.C.C., Yoshikawa F.S.Y., Pietrobon A.J., Sato M.N. (2018). Role of histamine in Modulating the Immune Response and Inflammation. Mediat. Inflamm..

[49] Sim Y.-B., Park S.-H., Kim S.-S., Kim C.-H., Kim S.-J., Lim S.-M., Jung J.-S., Ryu O.-H., Choi M.-G., Suh H.-W. (2014). The modulatory Role of Spinally Located Histamine Receptors in the Regulation of the Blood Glucose Level in D-Glucose-Fed Mice. Korean J. Physiol. Pharmacol..

[50] Sharma N., Sim Y.-B., Park S.-H., Kim S.-S., Lee J.-R., Jung J.-S., Suh H.-W. (2015). Effect of histamine receptors agonists or antagonists administered intracerebroventricularly and intrathecally on the blood glucose level in immobilization stress model. Anim. Cells Syst..

[51] Vallon V., Platt K.A., Cunard R., Schroth J., Whaley J., Thomson S.C., Koepsell H., Rieg T. (2010). SGLT2 mediates Glucose Reabsorption in the Early Proximal Tubule. J. Am. Soc. Nephrol..

[52] Nair S., Wilding J.P.H. (2010). Sodium glucose Cotransporter 2 Inhibitors as a New Treatment for Diabetes Mellitus. J. Clin. Endocrinol. Metab..

[53] Rafieian-Kopaei M., Nasri H. (2015). Diabetes mellitus and renal failure: Prevention and management. J. Res. Med. Sci..

[54] Peng H., Wang J., Ye X.Y., Cheng J., Huang C.Z., Li L.Y., Li T.Y., Li C.W. (2019). Histamine H_4_ receptor regulates IL-6 and INF-γ secretion in native monocytes from healthy subjects and patients with allergic rhinitis. Clin. Transl. Allergy.

[55] Triggiani M., Gentile M., Secondo A., Granata F., Oriente A., Taglialatela M., Annunziato L., Marone G. (2001). Histamine induces Exocytosis and IL-6 Production from Human Lung Macrophages Through Interaction with H1Receptors. J. Immunol..

[56] Morimitsu A., Sumigama S., Kotani T., Mano Y., Suzuki K., Araki-Taguchi M., Yamamoto E., Hayakawa H., Okada M., Itakura A. (2010). Histamine stimulates Interleukin-6 Production through Histamine H1 Receptors in Human Amnion Cells. Gynecol. Obstet. Investig..

[57] Li Y., Chi L., Stechschulte D.J., Dileepan K.N. (2001). Histamine-induced Production of Interleukin-6 and Interleukin-8 by Human Coronary Artery Endothelial Cells Is Enhanced by Endotoxin and Tumor Necrosis Factor-α. Microvasc. Res..

[58] Park I.-H., Um J.-Y., Cho J.-S., Lee S.H., Lee S.H., Lee H.-M. (2014). Histamine promotes the Release of Interleukin-6 via the H1R/p38 and NF-κB Pathways in Nasal Fibroblasts. Allergy Asthma Immunol. Res..

[59] Desai P., Thurmond R.L. (2011). Histamine H_4_ receptor activation enhances LPS-induced IL-6 production in mast cells via ERK and PI3K activation. Eur. J. Immunol..

[60] Su H., Lei C.-T., Zhang C. (2017). Interleukin-6 signaling Pathway and Its Role in Kidney Disease: An Update. Front. Immunol..

[61] Anbar H.S., Shehatou G.S., Suddek G.M., Gameil N.M. (2016). Comparison of the effects of levocetirizine and losartan on diabetic nephropathy and vascular dysfunction in streptozotocin-induced diabetic rats. Eur. J. Pharmacol..

[62] Verta R., Grange C., Gurrieri M., Borga S., Nardini P., Argenziano M., Ghè C., Cavalli R., Benetti E., Miglio G. (2019). Effect of bilastine on Diabetic Nephropathy in DBA2/J Mice. Int. J. Mol. Sci..

[63] Qi Z., Fujita H., Jin J., Davis L.S., Wang Y., Fogo A.B., Breyer M. (2005). Characterization of susceptibility of Inbred Mouse Strains to Diabetic Nephropathy. Diabetes.

[64] Kitada M., Ogura Y., Koya D. (2016). Rodent models of diabetic nephropathy: Their utility and limitations. Int. J. Nephrol. Renov. Dis..

[65] Sohara E., Rai T., Miyazaki J.-I., Verkman A.S., Sasaki S., Uchida S. (2005). Defective water and glycerol transport in the proximal tubules of AQP7 knockout mice. Am. J. Physiol. Physiol..

