# Supplementary material for: The Interplay between Histamine H4 Receptor and the Kidney Function: The Lesson from H4 Receptor Knockout Mice
Source: Biomolecules. 2021 Oct 15;11(10):1517. doi: 10.3390/biom11101517 (PMC8533779; doi:10.3390/biom11101517)
Supplement: Supplementary file 1 [file biomolecules-11-01517-s001.zip › biomolecules-1318500-supplementary.pdf]

Table S1. Row in vivo data: $H_4R^{-/-}$ mice weight

Group	Starting age	Day								
		T0	15	30	45	60	75	90	105	116
Control	1 11-12 WEEKS	27.95	29.08	29.29	29.30	29.93	32.70	31.66	31.10	31.19
		26.13	28.16	29.10	28.56	28.68	31.27	33.10	31.44	31.90
		25.79	27.89	29.18	28.72	29.08	31.69	32.08	31.79	32.31
	7-8 WEEKS	21.77	22.90	23.83	24.55	25.08	26.14	27.57	26.40	28.40
		19.36	21.75	24.24	23.99	23.29	25.41	25.79	26.38	26.89
		19.14	20.10	22.42	21.97	21.99	23.89	25.20	24.70	24.48
		22.53	24.30	25.89	26.55	26.65	28.74	28.80	28.84	30.45
	5-6 WEEKS	22.55	23.07	24.65	24.82	24.65	26.87	27.71	28.15	28.40
		23.41	25.53	27.60	27.71	28.22	31.06	31.56	31.53	32.15
Streptozotocin	11-12 WEEKS	26.68	26.58	27.72	26.79	25.39	27.99	26.66	23.94	24.92
		25.24	25.02	25.46	25.70	23.79	26.31	24.03	22.99	24.29
		25.07	26.56	26.17	27.10	25.67	27.99	27.68	26.36	27.19
		24.91	24.21	26.24	25.42	24.23	27.40	25.56	24.92	26.37
		26.39	26.12	27.42	27.50	25.73	27.54	27.44	26.72	27.01
		24.00	24.62	25.41	25.00	23.00	25.83	24.83	24.09	25.43
		26.2	26.47	28.42	27.99	26.57	28.39	28.58	27.86	27.62
	7-8 WEEKS	20.48	20.33	22.59	21.55	21.39	21.83	21.78	21.42	22.98
		21.91	22.38	23.58	22.73	21.35	21.19	21.91	21.62	22.14
		22.36	22.28	22.75	21.62	19.61	19.23	22.81	22.60	22.69
		23.53	23.58	24.79	24.69	22.99	25.20	25.43	25.00	26.07
		22.21	21.99	24.11	23.39	22.27	24.47	24.81	24.17	25.19
	5-6 WEEKS	23.40	23.01	23.36	25.08	23.22	26.14	26.56	24.27	24.40
		20.72	21.51	23.22	23.01	22.56	24.84	24.66	20.57	21.99
		21.32	22.64	23.43	24.27	23.12	25.72	24.64	21.44	22.44
	13-14 WEEKS	23.94	26.02	26.35	25.73	24.70	26.80	27.29	25.86	27.49
		25.11	25.46	26.52	25.72	24.50	26.45	27.03	26.31	26.33

Table S2. Row *in vivo* data: wild-type mice weight

Group	Starting age	Day								
		T0	15	30	45	60	75	90	105	116
Control	**1 11-12 WEEKS**	23.18	22.44	23.39	21.34	26.76	27.54	26.85	28.60	28.49
		21.88	22.30	24.41	25.38	28.03	28.74	28.37	30.77	28.19
		22.50	21.98	24.64	25.62	28.12	28.39	29.61	31.85	30.33
	**7-8 WEEKS**	20.46	22.34	23.09	21.24	25.56	26.76	27.54	28.37	30.77
		20.82	21.35	22.90	23.13	25.38	26.73	26.72	29.30	27.23
		20.86	22.47	25.24	25.26	28.95	29.57	29.70	31.71	29.51
		20.37	21.28	19.05	23,52	25.92	27.06	27.06	28.60	27.77
	**5-6 WEEKS**	19.81	21.16	20.29	22.13	23.13	24.68	25.15	25.37	25.25
		19.68	21.50	21.23	22.97	24.24	25.62	26.18	27.54	27.84
Streptozotocin	**11-12 WEEKS**	22.55	22.28	23.39	24.32	24.95	25.80	26.05	28.45	26.13
		22.41	20.38	20.93	21.91	23.29	24.11	24.11	26.03	24.02
		22.12	22.80	24.21	23.97	25.13	25.59	24.83	27.04	26.82
		22.96	21.51	22.97	22.54	23.34	24.19	23.46	26.58	24.62
		21.98	22.44	23.58	23.49	24.51	24.67	25.44	27.19	26.15
		21.33	22.26	24.48	25.99	27.07	27.13	27.20	29.60	28.02
		22.13	24.29	25.67	26.44	29.02	29.33	29.43	31.45	29.34
	**7-8 WEEKS**	21.11	21.50	21.63	19.33	22.63	24.64	25.12	26.48	25.28
		20.61	20.44	20.87	19.71	21.76	23.68	23.58	24.89	24.35
		20.65	18.98	19.32	17.04	22.44	24.22	24.29	24.98	24.47
		20.66	21.86	22.85	22.86	24.12	26.28	26.91	27.86	27.34
		21.21	21.99	24.11	23.39	22.27	24.47	24.81	24.17	25.19
	**5-6 WEEKS**	19.67	21.07	21.83	22.78	24.32	25.62	26.31	27.31	26.44
		19.77	18.49	19.20	19.31	20.72	22.03	21.52	23.28	21.63
		18.97	19.05	16.62	19.71	21.40	22.57	22.61	23.96	22.88
	**13-14 WEEKS**	25.55	24.72	26.59	27.75	28.69	29.98	30.34	31.22	30.52
		26.49	26.19	26.12	28.13	29.15	28.42	27.75	28.69	29.98

Table S3. Row *in vivo* data: $H_4R^{-/-}$ mice glycemia

Group	Starting age	Day								
		T0	15	30	45	60	75	90	105	116
Control	1 11-12 WEEKS	179	74	137	74	81	79	85	166	190
		132	75	106	118	153	103	121	143	119
		175	76	67	96	140	160	82	130	205
	7-8 WEEKS	90	84	94	96	89	63	139	181	134
		158	69	94	106	93	168	124	67	186
		232	46	150	137	76	105	189	198	139
		139	85	149	174	143	91	117	172	157
	5-6 WEEKS	134	67	153	156	175	191	135	178	153
		67	76	149	140	135	199	121	276	165
Streptozotocin	11-12 WEEKS	115	374	299	406	600	311	424	600	600
		131	218	181	204	240	393	600	543	600
		148	257	153	276	247	147	247	289	511
		126	147	255	434	489	600	600	600	600
		95	232	138	365	600	247	255	591	272
		124	340	169	412	600	547	433	600	600
		116	100	394	157	211	185	327	340	481
	7-8 WEEKS	179	148	381	199	256	187	419	600	600
		132	318	308	440	396	365	397	600	480
		175	250	282	308	403	569	600	574	456
		98	159	287	307	448	316	429	600	536
		116	148	145	94	132	217	324	483	478
	5-6 WEEKS	144	106	166	202	104	199	168	266	246
		126	261	316	361	336	600	600	454	600
		225	329	368	351	600	473	600	415	600
	13-14 WEEKS	131	118	155	123	155	212	378	220	393
		149	115	221	341	123	318	411	431	451

Table S4. Row *in vivo* data: wild-type mice glycemia

Group	Starting age	Day								
		T0	15	30	45	60	75	90	105	116
Control	1 11-12 WEEKS	165	124	139	196	99	154	117	123	89
		135	61	122		165	154	175	127	113
		164	124	162	180	186	129	163	165	134
	7-8 WEEKS	131	219	153	190	141	115	205	218	119
		148	86	162	127	125	194	143	130	70
			82	178	165	82		153	83	93
		115	111	126	101	163	141	116	85	132
	5-6 WEEKS	126	101	163	181	123	107	86	118	85
		144	145	190	115	111		151	130	142
Streptozotocin	11-12 WEEKS	179	270	198	413	274	215	270	252	336
		132	406	487	431	233	148	223	254	285
		175	140	230	210	123	107	86	118	85
		98	242	286	282	205	267	165	309	336
		129	297	395	423	193	252	256	263	230
		140	333	331	400	262	333	250	261	230
		127	209	427	399	374	259	421	600	370
	7-8 WEEKS	193	241	493	476	332	380	374	401	432
		204	492	439		506	440	550	600	600
		204	349	440	467	376	505	508	521	566
		90	121	366	430	339	203	367	600	600
		141	416	421	293	424		422	426	485
	5-6 WEEKS	130	436	481	600	333	247	459	344	400
		163	357	546	466	351	280	335	600	512
		95	230	419	235	367	370	478	542	433
	13-14 WEEKS	97	339	393	366	366	184	245	382	334
		188	190	261	174	268	128	167	334	376
